# A New Strategy of Chemical Photo Grafting Metal Organic Framework to Construct NH_2_-UiO-66/BiOBr/PVDF Photocatalytic Membrane for Synergistic Separation and Self-Cleaning Dyes

**DOI:** 10.3390/molecules28227667

**Published:** 2023-11-19

**Authors:** Lin Peng, Yong Shu, Luming Jiang, Weidong Liu, Guixiang Zhao, Rui Zhang

**Affiliations:** 1 PetroChina Research Institute of Petroleum Exploration & Development, Beijing 100083, China; 2 Key Laboratory of Oilfield Chemicals, China National Petroleum Corporation (CNPC), Beijing 100083, China; 3 College of Chemistry and Chemical Engineering, Southwest Petroleum University, Chengdu 610500, China

**Keywords:** photoinduced grafting method, MOF, visible-light photocatalysis PVDF, ultrafiltration membrane NH_2_-UiO-66/BiOBr/PVDF, mixed dyes

## Abstract

Photocatalytic membranes are typical multifunctional membranes that have emerged in recent years. The lack of active functional groups on the surface of membranes made of inert materials such as polyvinylidene fluoride(PVDF) makes it difficult to have a stable binding interaction with photocatalysts directly. Therefore, in this study, we developed a simple method to prepare NH_2_-UiO-66/BiOBr/PVDF(M_UB_) membranes for efficient dye treatment by grafting benzophenolic acid-functionalized NH_2_-UiO-66 onto the surface of membranes with photocatalytic properties under visible light irradiation using benzophenolic acid with photoinitiating ability as an anchor. The structural characteristics, photocatalytic properties, antifouling properties, and reusability of the composite membranes were investigated in subsequent experiments using a series of experiments and characterizations. The results showed that the benzophenone acid grafting method was stable and the nanoparticles were not easily dislodged. The M_UB_ composite membrane achieved a higher dye degradation efficiency (99.2%) than the pristine PVDF membrane at 62.9% within a reaction time of 180 min. In addition, the composite membranes exhibited higher permeate fluxes for both pure and mixed dyes and also demonstrated outstanding water flux recovery (>96%) after the light self-cleaning cycle operation. This combination proved to improve the performance of the membranes instead of reducing them, increasing their durability and reusability, and helping to broaden the application areas of membrane filtration technology.

## 1. Introduction

Even today, when people around the world are increasingly aware of environmental protection, there is still pollution and destruction of water resources, and the bad habits of industry and society are still deeply rooted, making it difficult to treat large volumes of industrial wastewater in an innovative way [1,2]. Dyes in water reduce the transparency of water by absorbing light, causing water hypoxia, destroying the ecological balance of water, and affecting the self-purification ability of water. Meanwhile, dyes have been confirmed to be carcinogenic and teratogenic, threatening life and health [3,4].

Many traditional treatment technologies have been used to treat polluted water, such as chemical oxidation, physical sedimentation, adsorption, coagulation, and biological treatment methods [5]. Membrane treatment technologies are widely used to effectively separate particulate matter, organic pollutants, and inorganic components in water due to their adjustable pore size, and they are gradually taking their place among water treatment methods because of their durability and continuous treatment to meet the cost-effectiveness considerations of dye wastewater purification processes [6,7]. The properties of membrane materials determine the performance of membrane filtration technology. Among them, PVDF membrane has excellent physical and chemical properties, as well as high temperature and corrosion resistance, and has attracted more and more attention in recent years [8]. As a result, PVDF membrane functions have been gradually developed for applications such as ultrafiltration, microfiltration, separation in membrane bioreactors, and cell membrane separation. However, the disadvantages of PVDF membranes are also obvious. The strong hydrophobicity of PVDF causes the membrane to be highly susceptible to contamination, which reduces service performance and service life [9]. Therefore, it is of great relevance to optimize the hydrophilic properties of PVDF membranes and to combine membrane filtration processes with other advanced water treatment technologies to give the membranes high performance and multi-functional characteristics [10,11].

Photocatalytic membranes combine both membrane separation and photocatalysis in an integrated system, which can expand the treatment effect and action surface, and the photocatalyst loaded on the membrane can be easily recycled without causing nanoparticle pollution [12]. In addition, this strategy can use natural light as the driving energy for catalyst activation, which is safe and green.

During the treatment process with the membrane, photocatalytic active groups (e.g., holes (h^+^), superoxide radicals (·O^2−^), and hydroxyl radicals (·OH)) are oxidized on the membrane surface to repel contaminants, thus reducing membrane contamination and significantly improving water flux. In this context, the advantages of photocatalytic membranes are highlighted in terms of simple separation and reuse of photocatalysts, mitigation of membrane contamination, and photocatalytic degradation of filter concentrates [13,14].

Most nanoparticles of conventional co-blended photocatalytic membranes are inevitably embedded in the native polymer and thus cannot be used to contact the reactant system for catalysis [15,16]. In order to expand the functionalization of membranes for applications, there is an urgent need to find new methods to stably link nanoparticles to the substrate membrane material [17]. Benzophenone is widely used as a common photoinitiator and crosslinker in materials science because of its unique photochemical properties and weak impact on the environment [18]. The mechanism of action is roughly that benzophenone can reversibly form a double radical triplet state under UV light irradiation, extract hydrogen atoms from the C-H bond in the material, and subsequently compound to form a stable covalent bond [19,20]. Notably, this photo-grafting approach has been applied to modify the surfaces of electrodes, photocatalysts, and biosensor materials and is also effective for PVDF [21].

In this study, we prepared the BiOBr/PVDF membrane by the in-situ deposition method first. Different from the simple physical mixing method, this method can better disperse BiOBr on PVDF. Then, we used 4-benzoylbenzoic acid as a bridge structure to graft the MOF material (NH_2_-UiO-66) onto the modified BiOBr/PVDF membrane under visible light irradiation. Through this photo-graft synthesis method, NH_2_-UiO-66 can be combined with the BiOBr/PVDF membrane by chemical bond, providing a bridge for electron transfer and thus improving the efficiency of photoelectric conversion. Meanwhile, the combination of NH_2_-UiO-66 and BiOBr/PVDF membrane is more stable, and the porosity of BiOBr/PVDF membrane will not be scaled and blocked during the modification process. The functionalized membranes were subsequently characterized by a series of scientific characterizations to confirm the feasibility and stability of this grafted structure and to assess its effect on the intrinsic membrane transport properties. The grafted NH_2_-UiO-66 acts as a synergistic photocatalyst with BiOBr on the membrane, giving the membrane a more powerful photocatalytic self-cleaning capability, which is demonstrated by recycling and self-cleaning experiments, and providing ideas for surface modification of inert materials and creating composite functional materials.

## 2. Results and Discussion

### 2.1. Structural Characteristics and Chemical Properties

**Figure 1:**
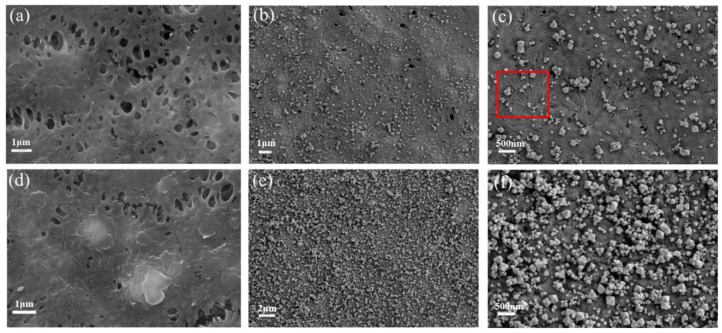
SEM image of (**a**) M_0_; (**b**,**c**) M_U0_; (**d**) M_B_; (**e**,**f**) M_UB_.

The scanning electron microscope was used to observe the surface and cross-sectional morphologies of membranes. We can clearly observe that the surface of the ungrown NH_2_-UiO-66 substrate membrane (Figure 1a,d is a normal porous shape, and the membrane assembled with benzophenolic acid has an octahedral shape of NH_2_-UiO-66 [22]. The red box in Figure 1c shows a certain spreading pattern, which we speculate may be a trace of benzophenolic acid growth on the membrane surface.

**Figure 2:**
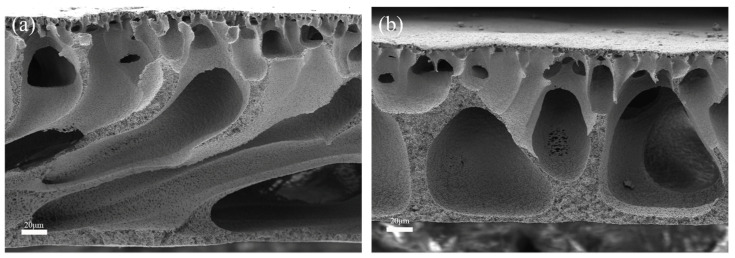
Cross-section image of (**a**) M_U0_ and (**b**) M_UB_.

The cross-sectional images still show the overall hollow porous structure, with the pristine PVDF membrane Figure 2a having a classical finger-like pattern and the skeletonized structure of the BiOBr/PVDF membrane Figure 2b being consistent with the cross-sectional structure reported between. Meanwhile, by comparing the two cross-sectional images, it can be seen that the pore surface of the membrane is very smooth after the photografting process. In addition, no bumps or dents occurred, indicating that the entire surface photoinitiation process of benzophenolic acid does not change the internal structure of the membrane and does not cause damage to the basic properties of the membrane [23].

**Figure 3:**
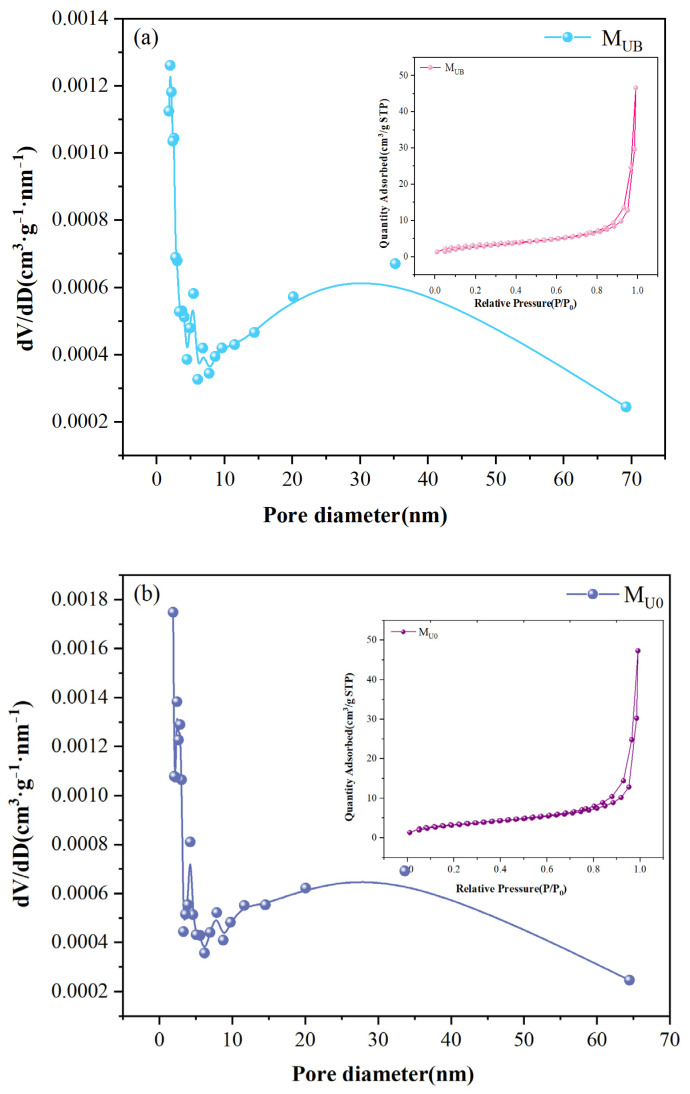
(**a**,**b**) are the corresponding pore size distributions of M_UB_ and M_U0_, respectively.

N_2_ adsorption-desorption measurements were performed on the membranes to evaluate the specific surface area and pore diameter distribution of M_UB_ and M_U0_. The specific surface areas of M_UB_ and M_U0_ were 11.709 m^2^/g and 12.566 m^2^/g, respectively. Both membranes exhibited type II-IV isotherms, as shown in the adsorption-desorption isotherm plot in Figure 3 [24]. The average pore diameter of the desorbed M_UB_ was 24.6117 nm, and that of M_U0_ was 23.3014 nm. Looking at the pore size distribution graphs again, the pore sizes of both membranes were mainly concentrated in the range of 2–30 nm with similar distribution forms, which indicates that although the substrate structures of the membranes were different, benzophenone could still graft the functionalized NH_2_-UiO-66 onto the substrate membrane uniformly without excessive overlap masking.

**Figure 4:**
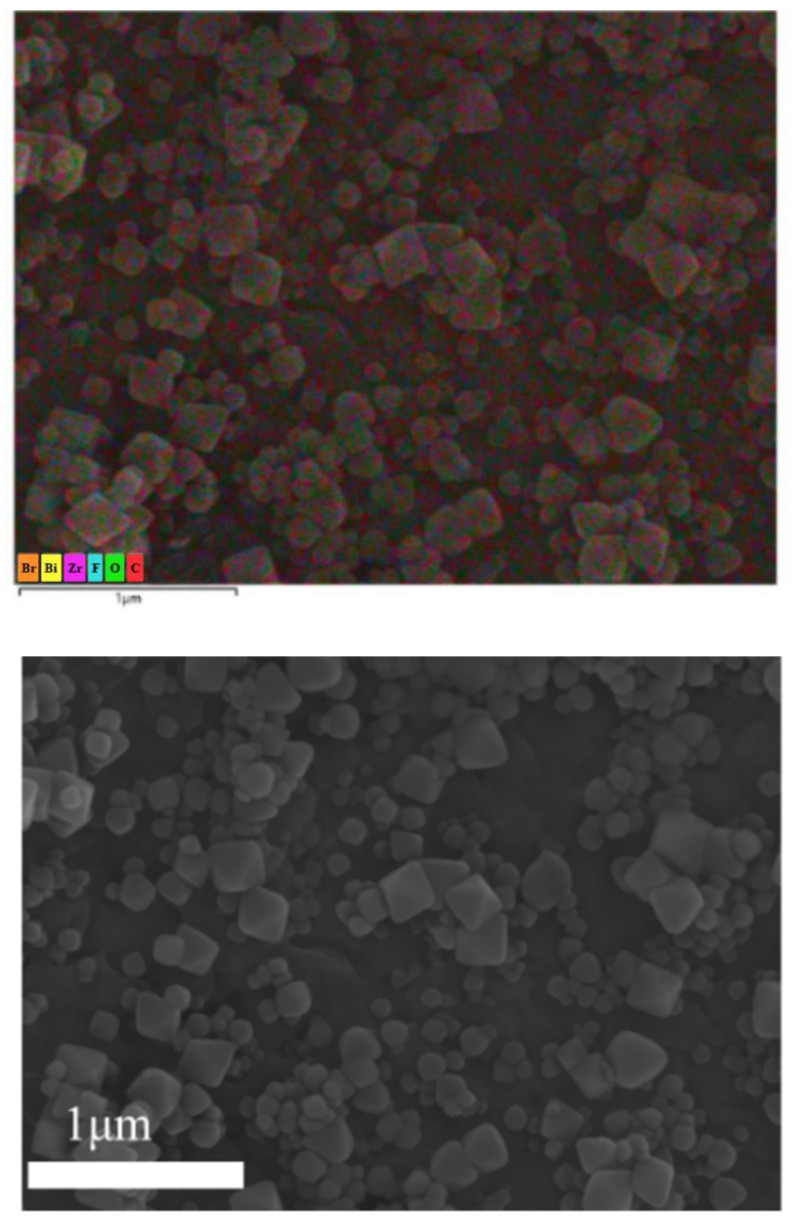

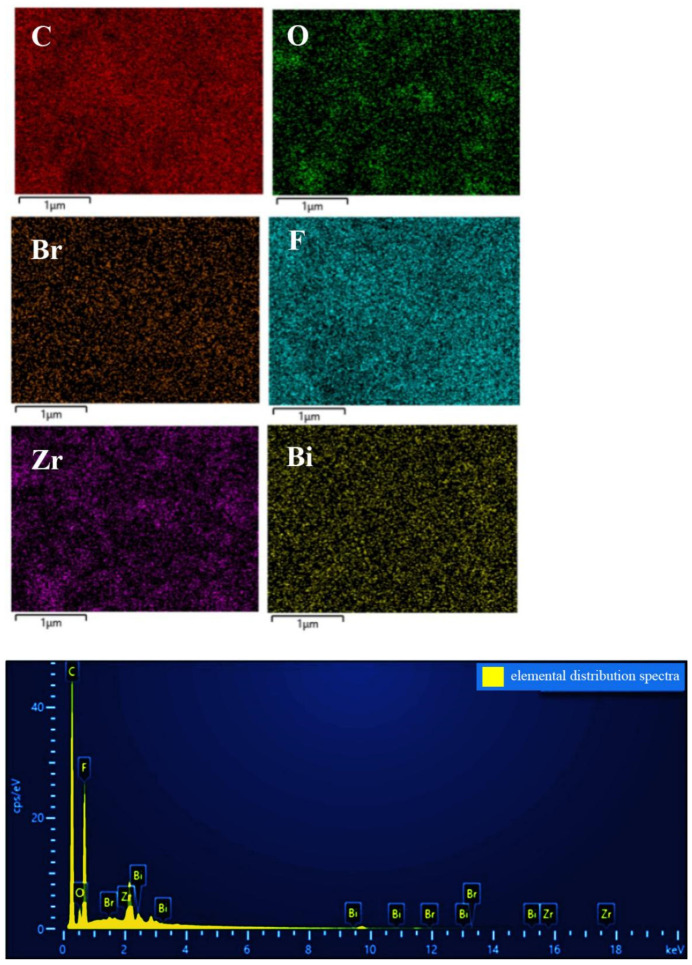
EDS elemental mapping scanning spectra of BP/ NH_2_-UiO-66/BiOBr/PVDF membrane.

The distribution and content of elements on the membrane surface were analyzed by EDS to verify whether NH_2_-UiO-66 was grown on the membrane surface. From the elemental distribution spectra (Figure 4), the typical elements C, F, Bi, and Br of the BiOBr/PVDF membrane are still present and uniformly distributed on the membrane surface, and the O element and characteristic element Zr contained in NH_2_-UiO-66 are more prominent in the octahedral distribution [25]. Meanwhile, the comparison of the elemental composition in Table 1 also shows that the mass percentages of C and O elements of M_UB_ increase and the remaining elements decrease compared to M_B_, which is due to the involvement of NH_2_-UiO-66 and benzophenolic acid in the system increasing their C and O elements, which verifies to some extent the grafting of octahedral NH_2_-UiO-66 by benzophenolic acid on BiOBr/ PVDF membrane [26].

**Figure 5:**
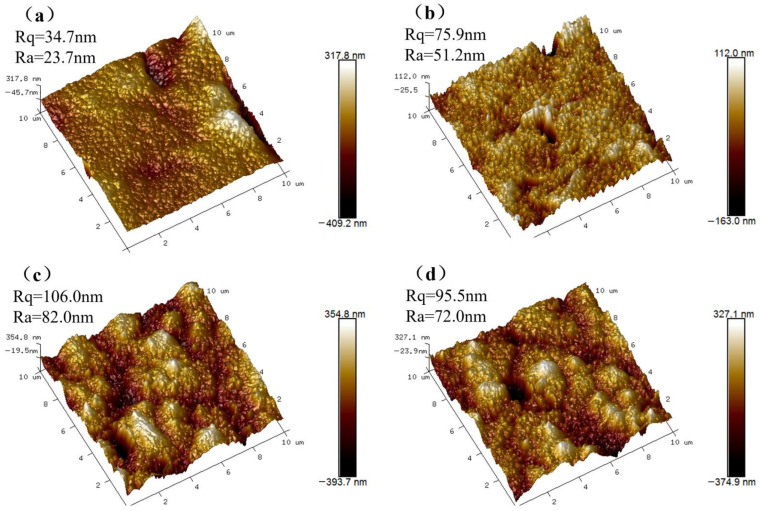
The AFM images of (**a**) M_B_; (**b**) M_0_; (**c**) M_UB_; (**d**) M_U0_.

The roughness of the membrane surface can be observed by atomic force microscopy [27]. In Figure 5, the bright parts represent bumps, and the gray parts represent depressions. In addition, we also labeled the average surface roughness (Ra) and mean square roughness (Rq) of the membranes in three-dimensional surface profiles, which, combined with the light and dark variations of the graphs and the data, can be used to infer the flatness of the membrane surfaces more intuitively. The average roughness of M_B_ (Ra, 23.7 nm) is smaller than that of M_0_ membrane (Ra, 51.2 nm), which is the smoothest and flattest among the M_B_, M_0_, M_UB_, and M_U0_ membranes, probably because the deposition of BiOBr in situ during phase transformation improves the hydrophilicity of the membrane, and the rate of phase separation of the casting solution in water is then slowed down, which weakens the speed competition difference between non-solvent phase separation and membrane solidification and reduces small ripple-like solid traces on the membrane surface and improves the smoothness of the membrane surface [28]. Due to the loading of NH_2_-UiO-66 on the membrane surface and its relatively random growth points, the roughness of the membrane increased substantially even with the M_B_ membrane as the substrate. However, it increases the permeability of the membrane, the increase in surface roughness also represents an increase in filtration area [29], the uniform peak distribution forms a good unity with the EDS mapping results, the retention of M_UB_ and M_U0_ can be predicted to be no worse, and there are many photocatalytic active sites available.

**Figure 6:**
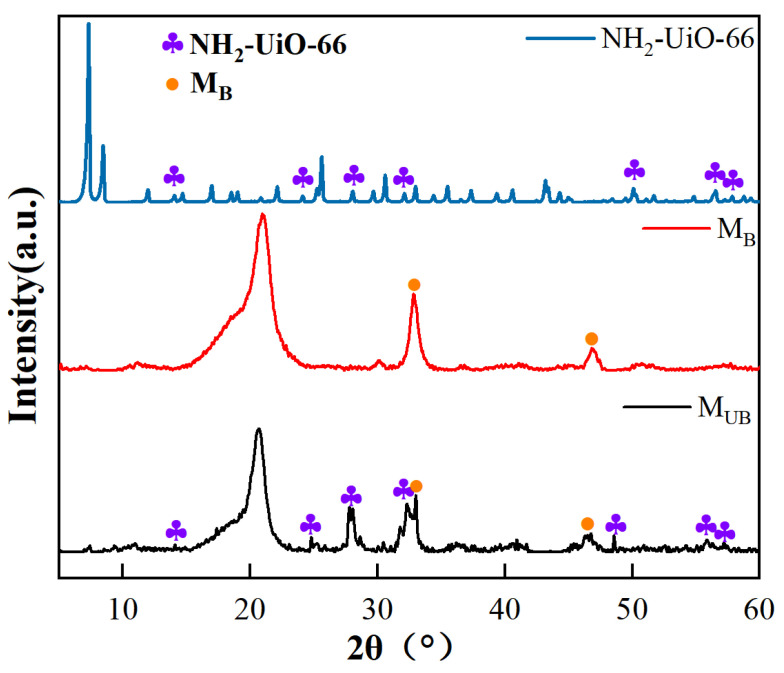
XRD diffractograms of NH_2_-UiO-66, M_B_, and M_UB_.

The composite NH_2_-UiO-66/BiOBr/PVDF membrane was characterized using XRD analysis, as shown in Figure 6. Since the PVDF membrane is not highly crystalline [30,31], the M_B_ and M_UB_ images are noisy, but the diffractogram pattern of the M_UB_ membrane is still found to combine some diffraction peaks of both M_B_ and NH_2_-UiO-66 monomer, indicating the presence of some microcrystalline sites from NH_2_-UiO-66 monomer and BiOBr on the polymer structure [32]. For example, near the diffraction angle of 32.90°, the M_B_ membrane is originally a single peak of moderate intensity, and the NH_2_-UiO-66 monomer has two small peaks. After the photo-grafting of the composite NH_2_-UiO-66, the M_UB_ diffractogram shows a split double peak of moderate intensity. The rest of the observed faint peaks can be related to the photocatalyst uniformly dispersed on the membrane, which can indicate, to some extent, the grafting of NH_2_-UiO-66 onto the BiOBr/PVDF substrate membrane via benzophenolic acid.

**Figure 7:**
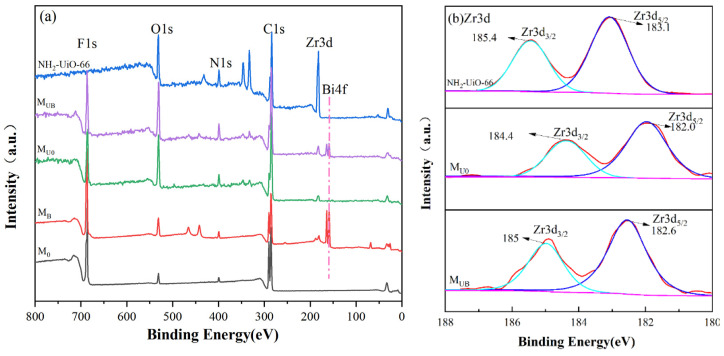
XPS spectra (**a**) survey; (**b**) Zr3d.

The XPS technique was used to determine the composition of membranes, and the corresponding broad and high-resolution spectra are shown in Figure 7. In the total spectrogram, the spectra demonstrate all the expected elements in the composite structure. For example, the zirconium element unique to NH_2_-UiO-66 is present in M_U0_ and M_UB_ membranes, and the Bi element unique to BiOBr is present in M_B_, M_U0_, and M_UB_ membranes, which explains to some extent the combination of the material and the membrane. Zr3d splits into two Gaussian-like orbitals, Zr3d5/2 and Zr3d3/2, and the Zr3d5/2 and Zr3d3/2 binding energies of M_UB_ are 182.6 eV and 185.0 eV, which are shifted compared to the peak of the NH_2_-UiO-66 monomer, indicating a change in the chemical environment of Zr, proving that NH_2_-UiO-66 is attached to the membrane by chemical bonding to the membrane rather than a mere physical stacking [33,34,35,36]. Meanwhile, the F1s and C1s of all the samples are 688.6 eV and 290.2 eV, respectively, and there is no change, which indicates that the chemical environment of C and F is not affected in the process of sample preparation. Combined with the previous XPS spectra of Zr, it can be proven that the composite of M_UB_ material is successful.

### 2.2. Membrane Performance Evaluation

#### 2.2.1. Hydrophilicity and Permeability

**Figure 8:**
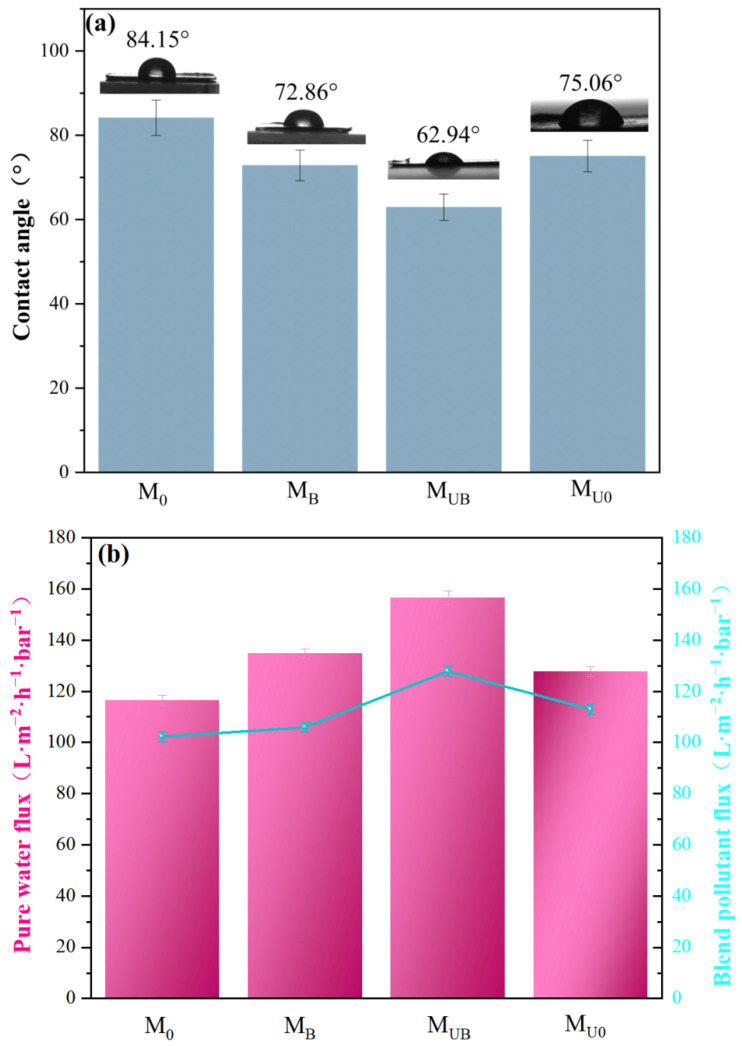
(**a**) Water contact angle of composite membrane; (**b**) pure water flux and mixed dye flux.

The hydrophilic properties and permeability of membranes can generally be assessed by their contact angle (CA) and flux [37]. In order to obtain higher accuracy of experimental results, five different positions on the membrane were selected for detection; each detection time was 60 s; and finally, the average value was taken as the detection result. As shown in Figure 8a, the normal PVDF membranes prepared by the conventional phase conversion method had severe surface hydrophobicity, and the contact angle to water of M_0_ is 84.15°. Compared with M_0_, the contact angles to water of M_B_, M_UB_, and M_U0_ all decreased, which were 72.86°, 62.94°, and 75.06°, respectively. The contact angle of M_UB_ was smaller than that of M_B_, and the contact angle to water of the NH_2_-UiO-66-loaded membrane was reduced to some extent, probably because NH_2_-UiO-66 preferentially contacted water with and the extended spatial structure provided the initiation effect, which improved the hydrophilicity.

Meanwhile, the water flux and mixed dye flux of M_0_, M_B_, M_UB_, and M_U0_ membranes at atmospheric pressure were measured by an ultrafiltration unit (Figure 8b). The fluxes of the four membranes M_0_, M_B_, M_UB_, and M_U0_ were 116.4, 134.7, 156.5, and 127.8 L·m^−2^·h^−1^⋅bar^−1^ for pure water and 102.2, 105.9, 127.8, and 112.9 L·m^−2^·h^−1^⋅bar^−1^ for mixed dyes, respectively. The fluxes of the four samples tended to be the same regardless of whether the separation object was pure water or mixed dyes. Compared with M_0_, the flux of M_B_ was higher, possibly because a small quantity of BiOBr precipitated on the membrane surface as a matter of priority, resulting in a higher flux of membrane. M_UB_ had the smallest contact angle and the highest flux, which may be because NH_2_-UiO-66 was more easily grafted on the M_B_ substrate membrane, and thus a larger amount of NH_2_-UiO-66 on the surface of the M_UB_ membrane could provide the membrane with a larger surface roughness. In addition, abundant oxygen-containing groups and photocatalytic sites have significant van der Waals attraction, thus improving membrane permeability [38]. The modified membranes showed enhanced fluxes for both pure water and mixed dyes, which also indicated that the NH_2_-UiO-66 loading did not clog the membrane pores.

#### 2.2.2. Separation Performance

**Figure 9:**
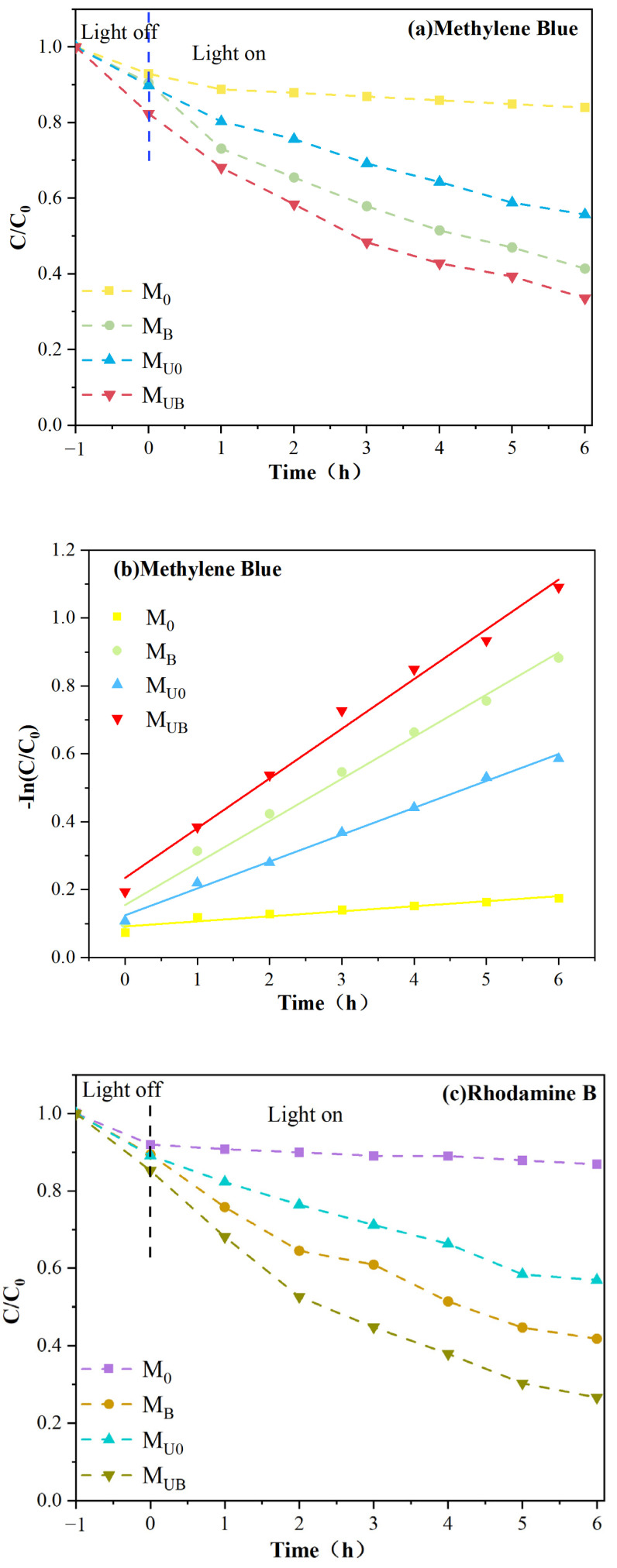

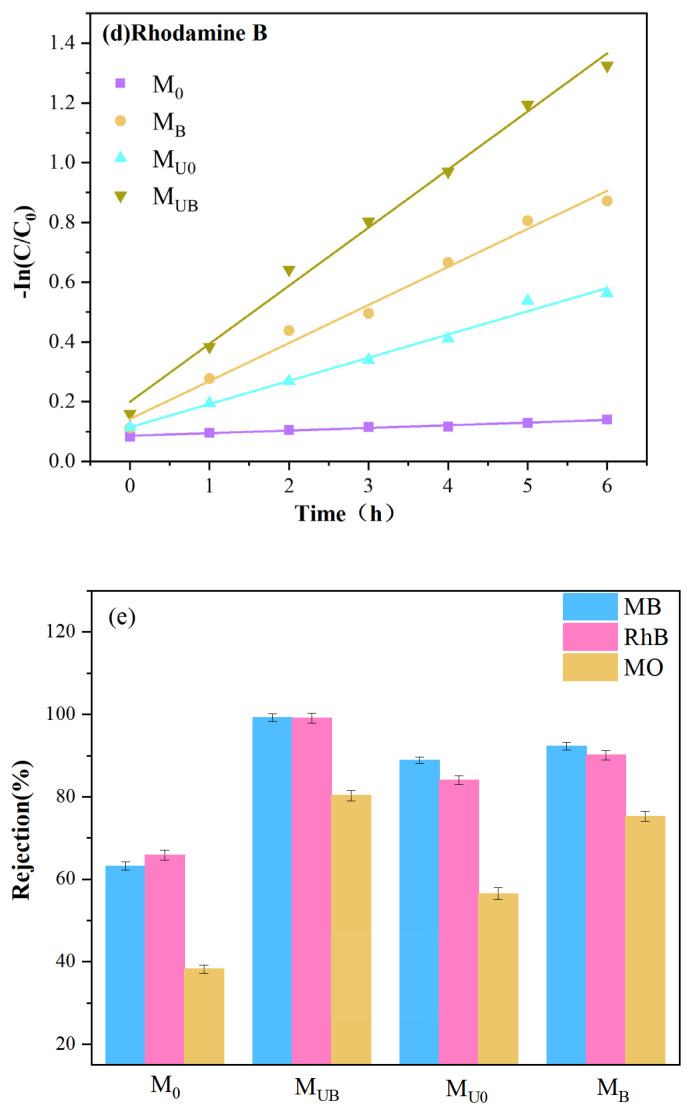
Degradation curves of (**a**) methylene blue (MB), (**c**) rhodamine B (RhB) for each type of membrane; (**b**,**d**) pseudo-first-order kinetic curves; (**e**) separation rate of the membrane for dyes (MB, MO, RhB).

The photodegradation performance of NH_2_-UiO-66/BiOBr/PVDF photocatalytic membranes was evaluated by a cyclic photocatalytic device. The variation of the concentration distribution (Ct/Co) of the tested membranes is shown in Figure 9a,c. Figure 9b,d shows the fitted curves of degradation kinetics of pseudo-primary kinetics for methylene blue and rhodamine B removal. Firstly, the membrane was adsorbed under dark conditions for 1 h to reach adsorption saturation, then the photocatalytic membrane was irradiated with the lamp turned on, and the pollutant concentration was measured by sampling every half hour. The experimental results showed that there was a small decrease in the dye’s concentration in all experimental groups during the dark adsorption phase [39]. This decrease could correspond to the van der Waals force attraction between the nanoparticles and oxygen-rich parts of the membrane surface and the charged pollutant molecules, and the adsorption could also occur in the mesoporous, microporous structure of the membrane itself [40]. The adsorption performance of the membranes grafted with NH_2_-UiO-66 was more pronounced because of their space-cage structure and larger specific surface area. After turning on the lamp for a period of time, the concentration of M_0_ dye without photocatalyst loading basically did not decrease, indicating that the pure PVDF membrane did not have any photocatalytic effect. The photocatalytic performance of the remaining three groups of membranes M_B_, M_U0_, and M_UB_ for methylene blue and rhodamine B after 6 h of light irradiation reached 58.6%, 44.3%, 66.4%, and 58.2%, 43.1%, and 73.4%, respectively. Moreover, the degradation rates of all the test samples for the pollutants were as follows: M_UB_ > M_B_ > M_U0_ > M_0_. The higher the rate constant, the faster the photocatalytic degradation rate was. It showed that the NH_2_-UiO-66/BiOBr/PVDF membrane possessed the highest photocatalytic performance among the four membranes.

The surface of the M_B_ membrane itself has in situ deposited BiOBr; photogenerated electrons can be transferred to the conduction band of BiOBr, leaving some holes, so it has certain photocatalytic performance, and the reason for the photocatalytic ability of M_UB_ is similar to that of M_B_ [41]. By grafting NH_2_-UiO-66 on the surface of M_B_, the photogenerated electrons can continue to transfer to the valence band of NH_2_-UiO-66, leaving a lot of holes to play a role in the oxidation ability, thus further enhancing the photocatalytic degradation efficiency. The modified membranes exhibited improved pollutant removal performance under visible light irradiation and reinforced the finding that photocatalysis dominates over adsorption processes in the photodegradation of pollutants. Under visible light irradiation, the modified films showed improved pollutant removal ability and showed that photocatalysis was more dominant in pollutant degradation than the adsorption process.

Secondly, separation efficiency is a crucial indicator for evaluating membrane performance. In order to evaluate the effectiveness of the membrane truthfully, this experiment used a mixture of MB, RhB, and MO to simulate sewage and measured the separation performance of the membrane. The mixed dye retention rate of the membrane was measured using a conventional static pressure filtration device, and the operating pressure of the membrane was always maintained at 0.1 MPa.

As shown in Figure 9e, the retention of mixed dyes by M_0_ was 62.9%, 66.7%, and 37.4%, respectively, and the retention of M_B_ (92.3%, 90.1%, and 75.3%) was significantly higher for the modified membrane without NH_2_-UiO-66 grafting; after surface grafting with NH_2_-UiO-66, the retention of both M_UB_ (99.2%, 98.9%, and 80.7%) and M_U0_ (88.4%, 84.5%, and 56.5%) was increased compared to their respective substrate membranes, and the M_UB_ membrane was the most efficient membrane for separation. Macroscopically, flawless surfaces and uniformly distributed photocatalysts also have better value in removing pollutants from the membrane surface. This may be due to the highly hydrophilic sites on the modified membrane surface exerting repulsive forces on stains of different polarity, thus improving the separation efficiency of the contaminants. In addition, according to our previous studies [42], the surface potential of the membrane also has a certain effect on the degradation of dyes. The more negative the surface potential, the stronger the electron transport capacity and the higher the photocatalytic efficiency, which is also one of the reasons why M_UB_ has a high dye rejection of MB and RhB.

#### 2.2.3. Light Self-Cleaning Cycle Test

The anti-fouling and reusability of membranes are important criteria for evaluating their comprehensive performance. If the membrane is poorly resistant to contamination, contaminants in the wastewater (e.g., proteins and excess dyes) will deposit or adsorb pores or folds and plug the pores of membranes, which will reduce the penetration efficiency of the entire system, increase energy loss, and reduce membrane surface hydrophilicity and flux. Second, even if certain substances do not directly contaminate the membrane, excessive accumulation can provide a breeding ground for contaminants or bacteria that can damage the membrane [17]. Therefore, we evaluate the membrane’s light self-cleaning cycling performance by calculating the FRR and RFR of the membrane under light conditions (Table 2).

**Figure 10:**
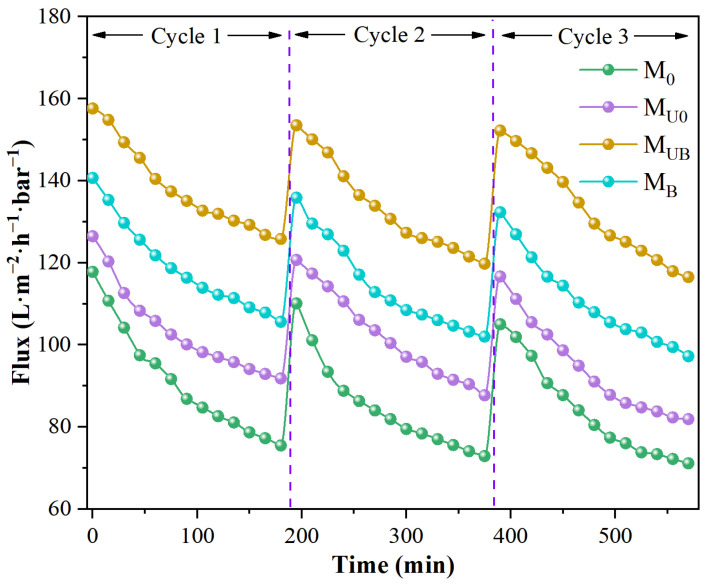
Membrane light self-cleaning cycle test chart.

In this study, three light self-cleaning cycles were conducted (Figure 10). Approximately 30 mg/L MB was used as the contaminant solution, and the membrane continuously retained the contaminant solution for 180 min per cycle, with samples taken every fifteen minutes for testing. After a full interception process, the membranes were removed, rinsed briefly with pure water, and then irradiated with a lamp for one hour to clean the membrane of dye residues. With the passage of the retention time, the thickness of the cake layer formed by the contaminants on the membrane gradually increased, and the dense cake structure caused the flux of all the membranes to decay to a certain extent, resulting in an overall quasi-steady state. In the first cycle, the initial fluxes of M_UB_, M_B_, and M_U0_ reached 158.1, 140.7, and 126.4 L·m^−2^·h^−1^·bar^−1^, respectively, all higher than the 117.8 L·m^−2^·h^−1^·bar^−1^ of the M_0_. The original membrane (M_0_) has the worst hydrophilicity and contamination resistance and the lowest flux, so grafting NH_2_-UiO-66 onto its surface with benzophenone can effectively improve the retention flux. At the same time, grafting NH_2_-UiO-66 onto the M_B_ substrate membrane, which has better properties, can further enhance the retention properties of the membrane. This indicates that grafting NH_2_-UiO-66 on the membrane using the light irradiation method can significantly improve the anti-pollution performance and flux of the membrane.

**Figure 11:**
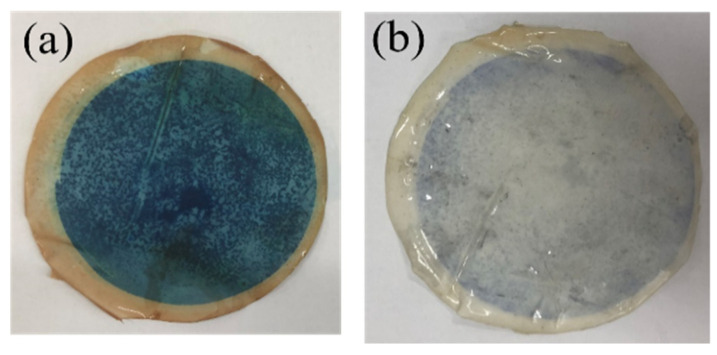

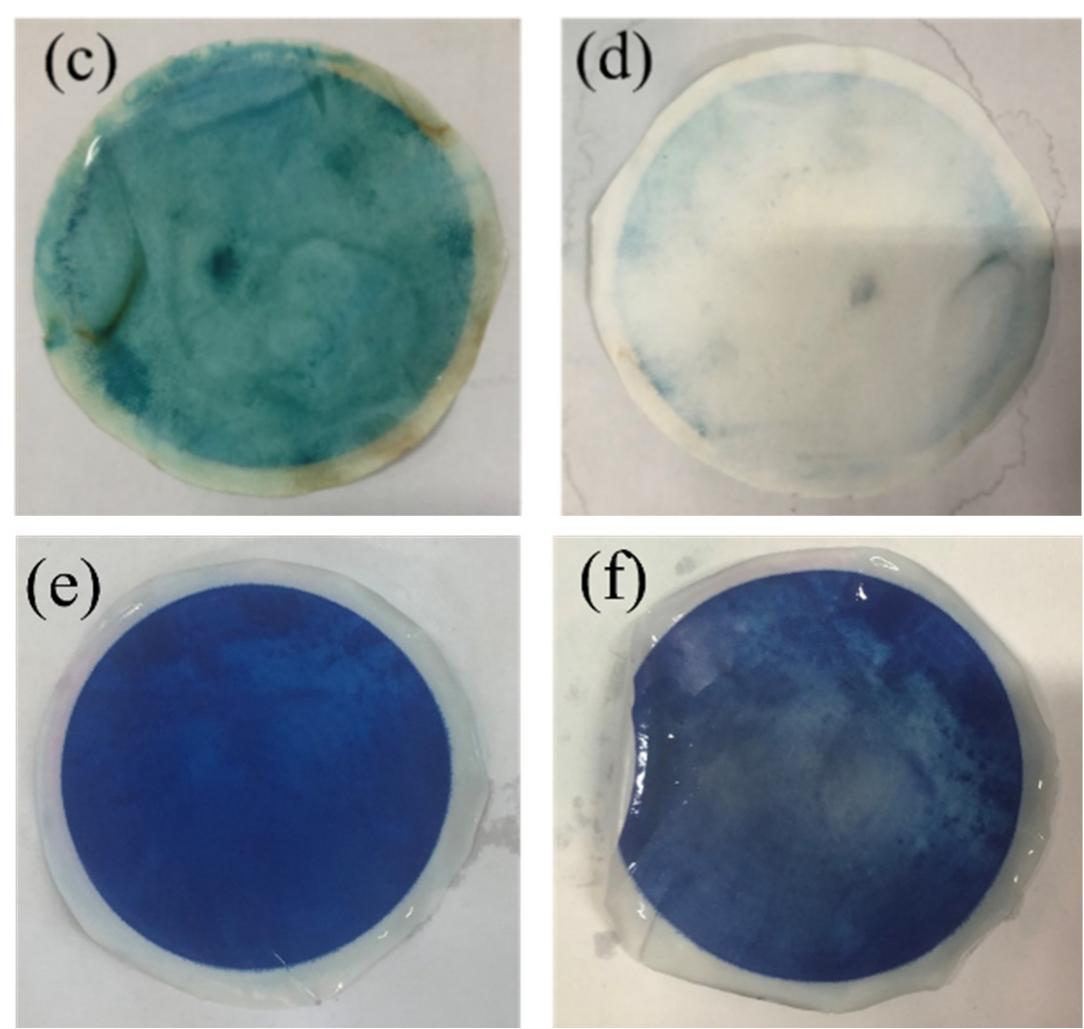
Membrane light self-cleaning cycle test chart: the images of the membranes M_B_ before (**a**) and after (**b**) light self-cleaning cycle, M_UB_ before (**c**) and after (**d**) light self-cleaning cycle, and M_U0_ before (**e**) and after (**f**) light self-cleaning cycle.

In addition, the flux recovery at the beginning of the second and third cycles reflects the light self-cleaning ability of the membrane, and the stronger the light self-cleaning ability, the better the initial flux recovery. The data show that the initial flux of the M_UB_ membranes basically did not decay in the three cycles, which is due to two main reasons: the anti-pollution ability of the membranes themselves, where dye molecules are not easily attached, and the high efficiency of NH_2_-UiO-66/BiOBr on the membrane surface to degrade the stubborn dye molecules attached to the membranes under light conditions [43]. Figure 11 shows the photocatalytic degradation ability of the membranes after three process cycles from a microscopic perspective, Figure 11a,b, Figure 11c,d, and Figure 11e,f corresponding to the images of the membranes M_B_, M_UB_, and M_U0_ before and after light self-cleaning, respectively. It can be seen intuitively that M_UB_ has the best light self-cleaning effect, and the methylene blue on the membrane surface is basically degraded. M_B_ has the second-best effect, and M_U0_ has only the NH_2_-UiO-66 monomer alone on the surface, and its monomer does not have an effective photocatalytic effect on the dye, so the photocatalytic effect of the membrane is also basically non-existent. The results showed that the prepared photocatalytic composite membranes, especially the NH_2_-UiO-66/BiOBr/PVDF membrane, have excellent long-lasting photocatalytic ability, an excellent decolorization effect, and a self-cleaning effect.

The size of the FRR value is positively correlated with the flux after self-cleaning and reflects the self-cleaning ability of the membrane, while the RFR value reflects the resistance of the membrane to contamination. The smaller the RFR value, the less the flux of the membrane decays, and the flux can be maintained at a high level. The calculation results for FRR and RFR are shown in Table 2. From the macroscopic point of view, all the membranes have a certain degree of flux reduction after repeated use; the more hydrophilic M_UB_ and M_B_ have a smaller decay than pure M_0_, and M_UB_ and M_B_ have a stronger flux recovery than M_U0_ and M_0_. M_UB_ and M_B_ have less decay than the pristine membrane M_0_ because of their better hydrophilicity, and the dyes and other impurities in the wastewater are less likely to adhere and block the membrane pores. The flux recovery after light self-cleaning is also stronger than that of M_U0_ and M_0_ because M_UB_ and M_B_ have photocatalytic ability and light can degrade the stubborn dye molecules remaining on the membrane. In total, the M_UB_ has a higher FRR and a lower RFR than the rest of the membranes, which is consistent with the argument that benzophenolic acid can be used for the combination of membranes and nanoparticles.

**Figure 12:**
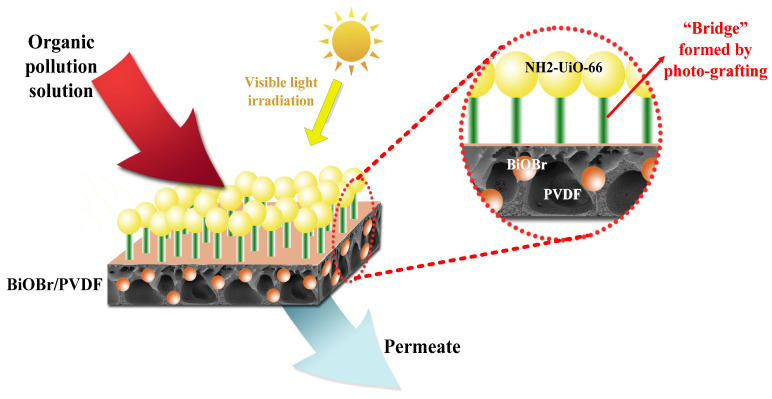
Diagram of the NH_2_-UiO-66/BiOBr/PVDF modified membrane retention process.

As can be seen from Figure 12, BiOBr is first uniformly dispersed on PVDF by in-situ deposition, and then NH_2_-UiO-66 is assembled on the BiOBr/PVDF membrane surface by chemical bond through photo-graft synthesis, forming a “bridge” for electron transfer. This structure can improve the efficiency and stability of electron transfer in the photocatalytic process, so as to significantly enhance the overall photocatalytic effect of the NH_2_-UiO-66/BiOBr/PVDF membrane. When organic pollutants pass through the membrane, they can be better purified.

## 3. Experimental Materials and Methods

### 3.1. Materials

Polyvinylidene fluoride (PVDF, FR904) powder was provided from Shanghai Tripartite New Materials Co. (Shanghai, China). Bismuth nitrate pentahydrate (Bi(NO_3_)_3_·5H_2_O), Polyvinylpyrrolidone(PVP), N,N-dimethylacetamide(DMAc), 2-[4-(2-hydroxyethyl)-1-piperazinyl]ethane sulfonic acid(HEPES), acetic acid, *N*-Hydroxysulfosuccinimide sodium salt(Sulfo-NHS), N1-((ethylimino)methylene)-N_3_,N_3_-dimethylpropane-1,3-diamine(EDC), 2-(N-Morpholino) ethanesulfonic acid monohydrate(MES), Benzophenone acid(BP), zirconium tetrachloride(ZrCl_4_), 2-aminoterephthalic acid(H_2_ATA), N,N-dimethylformamide(DMF), Methyl orange (MO), Rhodamine B (RhB), Methylene blue (MB), Sodium bromide(NaBr), ethanol and Sodium hydroxide (NaOH) were purchased from Kelong Chemical Co. Ltd., Chengdu, China. Deionized water (DI water, 18.25 MΩ/cm) was supplied from a PURELAB system (PURELAB Ultra Mk 2, ELGA). All analytical reagents were analytically pure and used directly without further purification.

### 3.2. Methods

#### 3.2.1. Preparation of NH_2_-UiO-66

Equal amounts (1 mmol) of H_2_ATA and ZrCl_4_ were added to 60 mL of DMF (99.8%), stirred until dissolved, followed by 7 mL of acetic acid (99%), ultrasonic for 10 min, and then reacted at 120 °C for 24 h. After removal, it was cooled to room temperature, cleaned alternately with DMF and methanol, and placed in a dryer at 60 ℃ for 48 h to obtain NH_2_-UiO-66 material.

#### 3.2.2. Synthesis of Benzophenone Functionalized NH_2_-UiO-66(BP-UiO)

Approximately 0.08 g of NH_2_-UiO-66 was dispersed in 10 mM of MES buffer solution (DI water, pH 6.0), then bath sonicated for 30 min to achieve a homogenous suspension. A total of 5 mL of each EDC (1.5 mM) and NHS (2.5 mM) solution prepared with MES as solvent was added continuously to the NH_2_-UiO-66 dispersion system. Adjust pH to 7.5 with sodium hydroxide/hydrochloric acid while continuously shaking, waiting for the conversion of the coo^−^ group of NH_2_-UiO-66 into amino-active esters [44]. Meanwhile, 10 mL of 5 mM ethylenediamine, 10 mM HEPES, and 0.5 mM NaCl solution was prepared as HEPES buffer at pH 7.5. Activated NH_2_-UiO-66 dispersion was mixed with HEPES buffer, and the system was kept shaking for 2 h, yielding amine-terminated NH_2_-UiO-66 nanoparticles [45].

**Figure 13:**
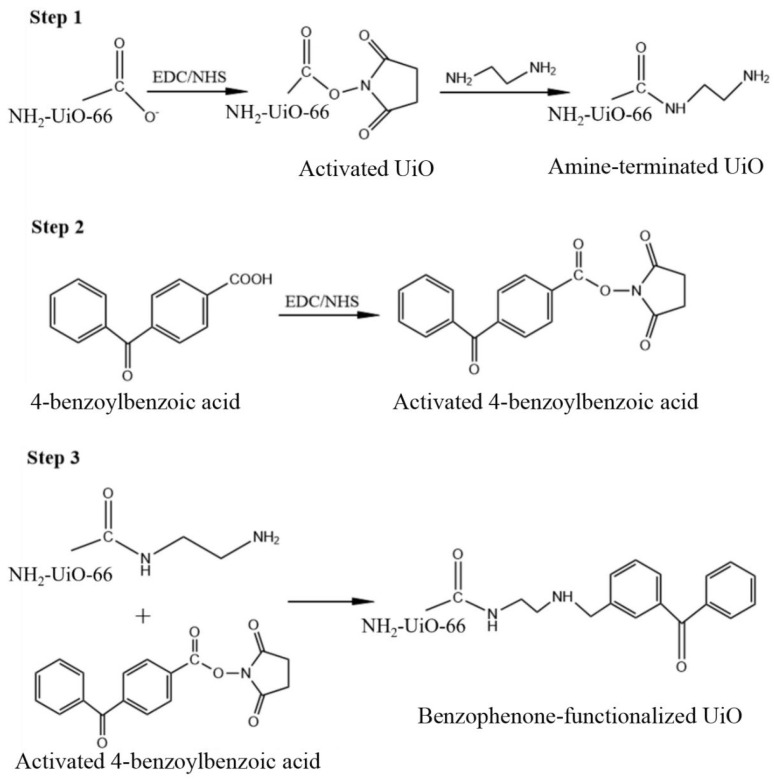
Synthesis mechanism of BP-UiO.

Approximately 4-Benzoylbenzoic acid (226.2 mg) was added to 70 mL of MES/methanol solution with a volume ratio of 1:1 and sonicated in a water bath for 1 h. Then, 2.25 mM of EDC solution and 3.75 mM of NHS solution were prepared using the MES/methanol mixture, respectively, and subsequently 5 mL of each was added to the 4-benzoylbenzoic acid solution, and stirring was continued for 30 min to allow the conversion of the natural carboxyl group of 4-benzoylbenzoic acid’s natural carboxyl group to be converted into a reactive ester. The previously obtained amine-capped NH_2_-UiO-66 was mixed with the activated 4-benzoylbenzoic acid, and then the pH was adjusted to 7.5 and gently stirred for 8 h. The amine-capped uio66 was attached to the reactive ester of the 4-benzoylbenzoic acid molecule by the formation of an amide bond. The synthesis process of BP-UiO is shown in Figure 13.

#### 3.2.3. Preparation of Functional NH_2_-UiO-66/BiOBr/PVDF Membrane

**Figure 14:**
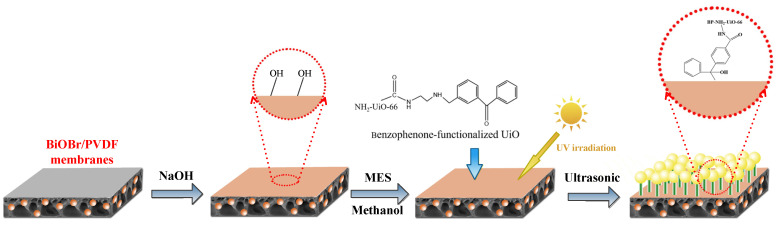
Flow chart for the preparation of the BP/NH_2_-UiO-66/BiOBr/PVDF membrane.

In this study, we selected the excellent-performance BiOBr/PVDF membrane prepared in previous studies as the substrate membrane [46]. The modified PVDF membranes were treated with 7.5 M NaOH at 70 °C for 3 h prior to the surface functionalization procedure to generate hydroxyl functional groups on the membrane surface. The modified PVDF membrane was contacted with a MES/methanol dispersion of BP-UiO. After soaking for 2 h, all membrane samples were irradiated with visible light in the air [13]. The membranes were bath sonicated for 10 min, then thoroughly rinsed three times with DI water and stored in DI water prior to use. The whole process diagram is shown in Figure 14.

In this study, M_0_ is referred to as the PVDF membrane, M_B_ is referred to as the BiOBr/PVDF membrane, and the membrane with NH_2_-UiO-66 photo-grafted on the pristine PVDF membrane and BiOBr/PVDF membrane is named M_U0_ and M_UB_, respectively.

### 3.3. Characterization of Membranes

Morphologies of membrane surface and cross-section were investigated using a scanning electron microscope (SEM, ZEISS Sigma 300, Jena, Germany). Brunauer-Emmet-Teller The Brunauer-Emmett-Teller method (BET, Micromeritics ASAP 2460, Atlanta, GA, USA) with nitrogen (N_2_) as a working gas was applied to observe the sorption behavior and the specific surface area of the photocatalytic membrane. Energy dispersive X-ray spectroscopy (EDS) analysis was performed using INCA and JSM-7500F at an acceleration voltage of 20 kV. Atomic force microscopy (AFM, Bruker Dimension Icon, Mannheim, Germany) was applied for the fabricated membranes’ surface roughness and topography observation with a scanned membrane area of 10 μm × 10 μm. X-ray diffractometers (XRD, PANaltical l, Dutch company, Amsterdam, The Netherlands) are used to analyze the crystal structure changes of the material using Cu target Kα, and the scanning angle and scanning speed are 2θ = 5–80° and 1°/min. X-ray photoelectron spectroscopy (XPS, Thermo Scientific ESCALAB Xi^+^, Waltham, MA, USA) was used to measure the chemical compositions of the membranes. The ultraviolet visible light spectrophotometer UV-Vis diffuse reflectance spectroscopy (UV–vis DRS) was used to analyze the optical absorption properties of samples. Operational details of some characterization items can be found in the literature [9,35,36,47,48].

### 3.4. Performance Evaluation of Membranes

#### 3.4.1. Hydrophilicity and Permeability

A dead-end ultrafiltration device (MSC300, Mosu Science, Shanghai, China) has been used to measure the filtration, which can reflect the permeability of the membrane. The effective filtration area of the membrane surface in this experiment was about 42 cm^2^. At the beginning of the experiment, the membrane was first placed under atmospheric pressure for half an hour to achieve a stable state. Then, the amount of deionized water penetrating the membrane was measured at a half-hour interval, and the flux can be obtained by Equation (1) [49].

(1) $$J_{w}=\frac{V}{A\times t}$$

where *J_w_* is the flux (L·m^−2^·h^−1^·bar^−1^), *V* (L) is the amount of penetrating the membrane during time *t* (h), and *A* (m^2^) is the effective surface area.

#### 3.4.2. Separation Performance

First, 30 mg each of methylene blue, methyl orange, and rhodamine were added to 1L of pure water to configure 30 mg/L of mixed dye wastewater. According to the specific absorbance of different samples, a visible spectrophotometer is used to determine the concentration of different permeate solutions. The absorbance of methylene blue, methyl orange, and rhodamine is 664 nm, 467 nm, and 554 nm, respectively. The separation rate (*R*) of the solutions can be calculated using Equation (2) [50]:

(2) $$R=(1-\frac{C_{p}}{C_{f}})\times 100\%$$

*C_p_* and *C_f_* are the concentrations of permeate and starting solutions in the samples, respectively.

#### 3.4.3. Evaluation of Flow Cycle Photocatalytic Performance

**Figure 15:**
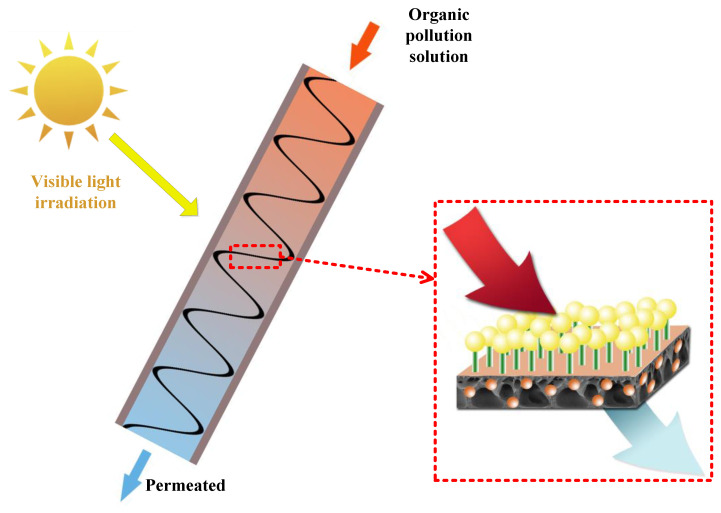
Diagram of a cyclic photocatalytic experiment.

A flow cycle device was designed to evaluate the photocatalytic effect of the prepared membranes under flowing water conditions. Figure 15 shows the flow-cycle photocatalytic device used in this experiment. Cut the membrane into long strips of 50 × 1 cm and lay them flat in the middle of the clear glass tube. A lamp light source is placed 30 cm above the glass tube, and a flow circulation pump is used below the glass tube to provide kinetic energy to promote the flow of liquid in the device. In this way, full contact between the membrane and the contaminant liquid can be achieved, simulating a realistic flow environment for flow photocatalysis experiments.

#### 3.4.4. Evaluation of the Photocatalytic Activity of the Fabricated Membranes

The photocatalytic activity of the prepared membranes was evaluated by measuring their degradation rates using a mixture of methylene blue, methyl orange, and rhodamine dye wastewater as the feed solution. First, 30 mg each of methylene blue, methyl orange, and rhodamine were added to 1 L of pure water to configure 30 mg/L of mixed dye wastewater, and then the membranes were cut into 50 cm × 1 cm long strips and placed in glass jars, and then the dye wastewater was added for cyclic broad photodegradation. Figure 3 is a schematic diagram of the photocatalytic experimental facility.

The experiment was conducted in an enclosed dark room topped with an ultraviolet lamp, and the distance between the lamp and the liquid to be measured was 10 cm. To ensure the analysis of the photocatalytic properties of the membrane, the membrane was soaked in the solution in dark conditions for two hours to achieve adsorption equilibrium before starting the test. This photocatalytic process was carried out for 6 h, with samples taken every 1 h to measure the removal of methylene blue, methyl orange, and rhodamine. Zero-level and pseudo-level kinetic models (Equations (3) and (4), respectively [51]) were used to calculate the kinetics of photocatalytic degradation of membranes.

C_0_ − C_t_ = k_0_t (3)

(4) $$\ln\;(\frac{C_{0}}{C_{t}})=k_{1}t$$

where C_0_ is the initial (mg/L) pollutant concentration and C_t_ is the time-specific (mg/L) pollutant concentration, k_0_ (min^−1^) and k_1_ (min^−1^) are the rate constants of the zero-order and pseudo-first-order kinetic models, respectively, and t is the run time (min).

#### 3.4.5. Long-Term and Light Self-Cleaning Performance

Three cycles of permeate flow and contaminant removal experiments were conducted to evaluate the manufactured membranes for their light self-cleaning ability and performance stability over time. To analyze the pollutant removal performance, the membranes were used for filtration experiments for 3 × 180 min, and the permeate flow was collected at a 30 min interval. After each cycle, the membrane is cleaned several times with deionized water and placed under a lamp for 1 h to ensure that the pollutants on the membrane are completely removed and that the membrane will not affect the experimental results in the next experiment.

#### 3.4.6. Antifouling Performance

In order to analyze the antifouling performance of the membrane, the penetration experiment is carried out using pure water (deionized water) and simulated polluted water, respectively, to carry out a comparative study. The specific experimental steps are in our previous work [46]. The antifouling of the membrane can be expressed in terms of the flux recovery rate (*FRR*) and relative flux decay rate (*RFR*), which can be obtained by using [Equation (5)]: [42]

(5) $$FRR=(\frac{J_{w2}}{J_{w1}})\times 100\%\;RFR=\left(\frac{J_{w1}-J_{w3}}{J_{w1}}\right)\times 100\%$$

where *J_w_*_1_ is the flux of the membrane at the beginning of the first cycle experiment, *J_w_*_2_ is the flux of the membrane at the beginning of the second cycle experiment, and *J_w_*_3_ is the flux of the membrane at the end of the first cycle experiment.

## 4. Conclusions

This study demonstrates the utilization of benzophenolic acid as an intermediate bridge structure, stably connecting the inert PVDF membrane with the metal-organic backbone through chemical bonding for the preparation of composite photocatalytic membranes comprising NH_2_-UiO-66/BiOBr/PVDF. The resulting composite membrane exhibits excellent intrinsic properties such as flux and retention rate, along with promising photocatalytic and self-cleaning capabilities. This bonding creates a stable electron transfer “bridge” between NH_2_-UiO-66 and the BiOBr/PVDF membrane, enabling efficient separation and degradation of dyes in wastewater, prolonging the membrane’s service life, and reducing costs, with practical significance for engineering practice. The intermediate bridging connection approach also provides insights for future research on combining inert membrane materials with inorganic or inert functional group materials. However, the method does have limitations, including cumbersome operation steps, restricted initiation conditions, and limited flat substitutes for bridge structures. We anticipate that future studies will build upon this foundation.

## Figures and Tables

**Table 1:** Chemical element contents of the pristine and M_UB_ membranes.

Membrane	Elemental Composition (wt%)					
	C	F	Bi	O	Br	Zr
M_B_	46.38	24.37	19.00	3.64	5.96	0
M_UB_	61.83	23.02	8.75	4.01	1.33	1.05

**Table 2:** Flux recovery ratio (FRR) and relative flux decay rate (RFR) of PVDF membranes (M_0_, M_B_, M_UB_, and M_U0_) in fouling and washing cycles.

Membrane	First Cycle		Second Cycle		Third Cycle	
	FRR	RFR	FRR	RFR	FRR	RFR
M_0_	85.3%	32.2%	83.4%	33.8%	79.2%	35.9%
M_B_	92.4%	24.9%	91.6%	25.1%	90.0%	26.5%
M_UB_	99.2%	20.2%	97.4%	22.0%	96.7%	23.5%
M_U0_	86.6%	26.7%	85.4%	27.4%	82.3%	29.8%

## Data Availability

Data are contained within the article.
